# A Cardiac Force Index Applied to the G Tolerance Test and Surveillance among Male Military Aircrew

**DOI:** 10.3390/ijerph18168832

**Published:** 2021-08-21

**Authors:** Kwo-Tsao Chiang, Min-Yu Tu, You-Jin Lin, Yi-Hsiang Hsin, Yu-Lung Chiu, Fang-Ling Li, Hsin-Hui Chen, Chung-Yu Lai

**Affiliations:** 1 Aviation Physiology Research Laboratory, Kaohsiung Armed Forces General Hospital Gangshan Branch, Kaohsiung City 820, Taiwan; charco66@gmail.com (K.-T.C.); du0807@yahoo.com.tw (M.-Y.T.); mmlieutenant@gmail.com (Y.-H.H.); 2 School of Public Health, National Defense Medical Center, Taipei City 114, Taiwan; long_ruth0624@yahoo.com.tw; 3 Department of Health Business Administration, Meiho University, Pingtung City 912, Taiwan; 4 Department of Life Sciences and PhD Program in Translational Medicine, National Chung Hsing University, Taichung City 402, Taiwan; 5 Institute of Medical Science and Technology, National Sun Yat-sen University, Kaohsiung City 804, Taiwan; 6 Graduate Institute of Aerospace and Undersea Medicine, National Defense Medical Center, Taipei City 114, Taiwan; youjinlin7@gmail.com; 7 Graduate Institute of Life Sciences, National Defense Medical Center, Taipei City 114, Taiwan; 8 Department of Psychiatry, Tri-Service General Hospital Beitou Branch, National Defense Medical Center, Taipei City 114, Taiwan; fanglinlee@gmail.com; 9 Department of Internal Medicine, Tri-Service General Hospital, National Defense Medical Center, Taipei City 114, Taiwan; orchid1319@gmail.com

**Keywords:** G force, anti-G straining maneuver, G tolerance, cardiac force index, relaxed G tolerance, straining G tolerance

## Abstract

Military aircrew are occupationally exposed to a high-G environment. A tolerance test and surveillance is necessary for military aircrew before flight training. A cardiac force index (CFI) has been developed to assess long-distance running by health technology. We added the parameter CFI to the G tolerance test and elucidated the relationship between the CFI and G tolerance. A noninvasive device, BioHarness 3.0, was used to measure heart rate (HR) and activity while resting and walking on the ground. The formula for calculating cardiac function was CFI = weight × activity/HR. Cardiac force ratio (CFR) was calculated by walking CFI (WCFI)/resting CFI (RCFI). G tolerance included relaxed G tolerance (RGT) and straining G tolerance (SGT) tested in the centrifuge. Among 92 male participants, the average of RCFI, WCFI, and CFR were 0.02 ± 0.04, 0.15 ± 0.04, and 10.77 ± 4.11, respectively. Each 100-unit increase in the WCFI increased the RGT by 0.14 G and the SGT by 0.17 G. There was an increased chance of RGT values higher than 5 G and SGT values higher than 8 G according to the WCFI increase. Results suggested that WCFI is positively correlated with G tolerance and has the potential for G tolerance surveillance and programs of G tolerance improvement among male military aircrew.

## 1. Introduction

Military aircrew are occupationally subjected to six axes of acceleration in flight [1]. Levels of downward inertial force can be quantified as gravity (+Gz, also called G force). Hydrostatic pressure produced by G force causes blood to flow toward the lower body region, and hemodynamic parameters are affected in hypergravity environments [2,3,4,5]. If G stress surpasses the tolerance, military aircrew will probably experience visual disturbances and even G-induced loss of consciousness (GLOC) that will extremely threaten the flight safety [6,7,8]. A tolerance test and surveillance in the human centrifuge is a prerequisite for military aircrew in many countries. Trainees can experience physiological impacts and perform an effective anti-G straining maneuver (AGSM) against GLOC. Activation of the cardiovascular system plays a crucial role in the tolerance of G force [9,10,11]. AGSM execution also simultaneously enhances cardiovascular performance under G load [12].

A number of reports have been conducted to investigate the relationship between G tolerance and cardiovascular index responses, such as heart rate (HR), mean arterial pressure, stroke volume, cardiac output, and total peripheral resistance [13,14,15,16]. As those factors are certainly correlated with each other, it is difficult to consolidate them into a simple one during practical training. In addition, the signal quality of those cardiovascular parameters was not good enough to be analyzed during a period of physical exertion or in high-G environment due to the limitations of the physiological monitoring machine. To date, there is no integrated cardiac function indicator that can be used to assess G tolerance on the ground before centrifuge training.

Recently, Hsiao et al. successfully applied a novel cardiac force index (CFI), which is calculated from the weight multiplying activity divided by HR and positively correlated to running performance [17,18,19]. From the literature review, we also summarized that weight is positively correlated with G tolerance [10,20]. In addition, our former findings identified that there is an inverse relationship between the G tolerance and HR on the ground [10,11]. As stated above, we therefore presumed that CFI might also be connected to G tolerance during the centrifuge training. We attempted to introduce the parameter CFI into the G tolerance assessment to address this concerning occupational safety issue.

The aforementioned cardiac function variable CFI was added to the G tolerance test for the first time. The main purposes of this study were to examine the association between cardiac function on the ground and G tolerance in the centrifuge and to further identify the difference in cardiac function between the different G tolerance groups. These findings will provide information on low-G tolerance subjects for initial aircrew selection before centrifuge training. Authorities could further establish a surveillance system of low-G tolerance, enact a program to improve the G tolerance of aircrew, and enhance flight safety.

## 2. Materials and Methods

### 2.1. Study Design and Participants

We organized this prospective longitudinal study to examine the association between the CFI on the ground and G tolerance in the centrifuge. Approval and permission for this study was granted by the ethics committee of the Institutional Review Board of Kaohsiung Armed Forces General Hospital in Kaohsiung City, Taiwan (No. KAFGH 109-001). Informed consent was provided by all the participants before the initiation of this study.

Participants were all male military aircrew members with no experience of high-G training and recruited from the Air Force Academy and National Defense Medical Center. All of them volunteered to join this study from April 2020 to January 2021 while attending high-G training in the Aviation Physiology Research Laboratory (APRL), Taiwan. APRL has conducted high-G training for military fighter pilots and crewmembers since 1996. The eligibility criteria for participants involved completing questionnaire items, physiological data monitoring, and high-G training.

### 2.2. Mobile Monitoring Device for Cardiac Function

The noninvasive mobile device used in the study was a BioHarness 3.0 (Zephyr Technology Corporation, Annapolis, MD, USA) equipped with a three-axis gyroscope and accelerometers to record the acceleration by piezoelectric technology. The main physiological parameters included the activity level, HR, and breathing rate. The activity level was calculated from the acceleration in the x, y, and z directions during the experiment. HR was measured by a conductive electrode sensor with the elasticity of the thoracic loop strap fit to the thoracic skin. The breathing rate was evaluated by a capacitive pressure sensor to evaluate the circumferential expansion and contraction of the chest wall. A shoulder strap was used to reduce the noise caused by displacement during physical activity [21]. The monitoring data could be displayed in real time by Bluetooth technology and stored in a memory to be exported for statistical analysis.

### 2.3. Study Protocol and Data Collection

**Figure 1:**
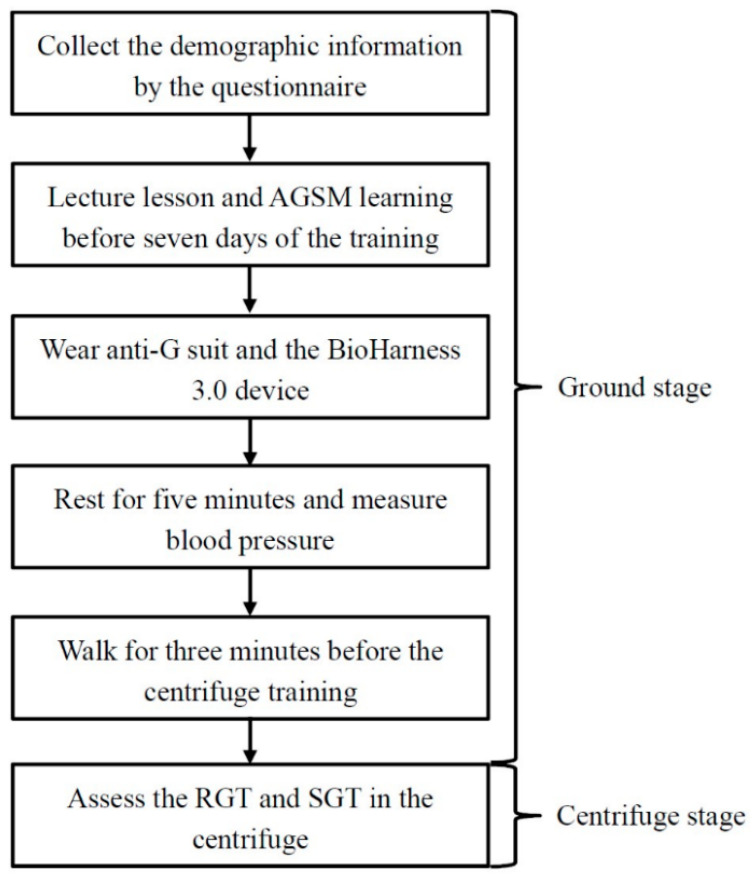
Study protocol.

The protocol in this study included a ground stage and a centrifuge stage (Figure 1). On the ground, we calculated the cardiac force function of the participants combined with a noninvasive wearable device. G tolerance was assessed in an artificial hypergravity condition as follows:

First, we used a questionnaire to collect participants’ demographic data, including age, gender, height, weight, body mass index (BMI), smoking status (yes: current smoker and ex-smoker, no), drinking status (yes: current drinker and ex-drinker, no), and exercise habits (yes, no). An aviation physiologist at APRL gave a lecture about acceleration physiology and demonstrated and described the techniques of AGSM for all participants. The techniques of the AGSM operation focus on forced respiration (also called the Valsalva maneuver) and lower body muscle strain. The participants learned to perform the AGSM properly and continued to master the AGSM before seven days of centrifuge training.

**Figure 2:**
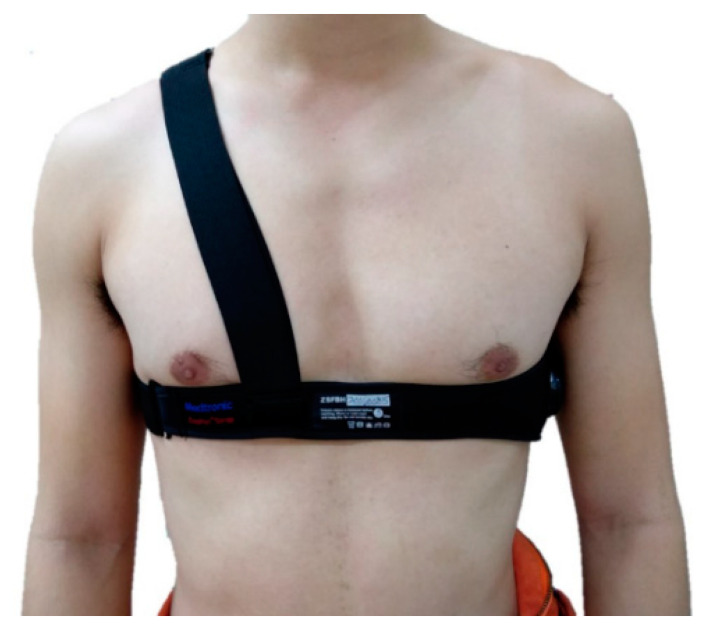
BioHarness 3.0 module attached to chest and shoulder straps.

On the day of the G tolerance test, they performed warm-up exercises and practiced the AGSM on the ground. Then, they wore five-bladder anti-G suits, and BioHarness 3.0 devices were used to record a variety of data of interest. The sensors were attached to the chest and shoulder straps and located at the left central armpit (Figure 2). The aviation physiologist turned on the BioHarness 3.0 devices worn by the participants to collect the data and examine their function and signal. After the participants rested in a seated position for five minutes, we measured their systolic blood pressure (SBP) and diastolic blood pressure (DBP) in a relaxed state by an Omron 1100U sphygmomanometer (Omron Healthcare Company, Taipei City, Taiwan). The aviation physiologist instructed them to squat down and stand up and then start walking at normal speed. After three minutes of walking, they squatted down and stood up again to complete the ground stage.

### 2.4. Centrifuge Stage

**Figure 3:**
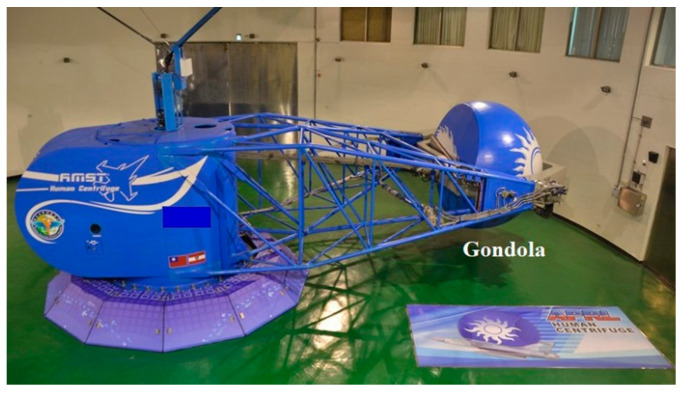
Human centrifuge in APRL.

In the 1990s, APRL was responsible for the procurement project of the human centrifuge manufactured by the French company Latécoère (Figure 3). The human centrifuge stationed in APRL is powered by a hydraulic system to simulate in-flight hypergravity conditions on the ground. Since 1996, APRL has provided centrifuge training for military aircrew to assess their G tolerance. The basic characteristics of the human centrifuge are a maximum training onset rate and G value of 6 G per second and 9 G, respectively. Participants wore five-bladder anti-G suits without inflation during the G tolerance test. After the ground stage, each participant sat down and fastened himself to a 13-degree reclined seat inside the gondola.

**Figure 4:**
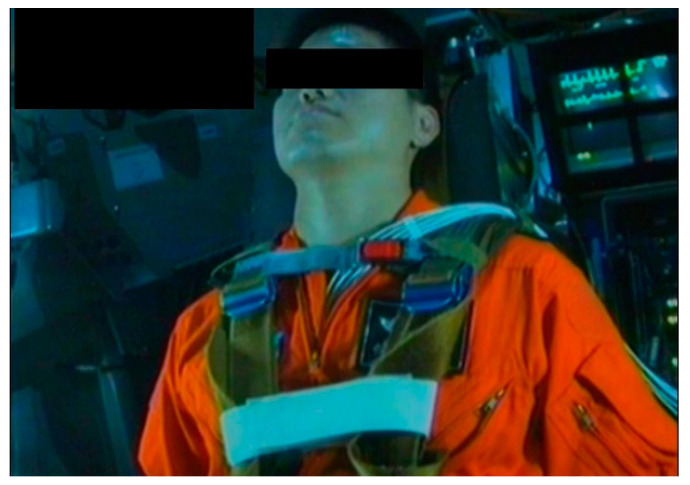
G tolerance test inside the gondola view.

Inside the gondola, participants had a two-minute seated rest to prepare for the G tolerance test in the 1.4 G environment. Thereafter, when they were ready to undergo the test, they must press the right-hand side stick on which there is a safety control system. The human centrifuge could be accelerated to assess the participants’ relaxed G tolerance (RGT) and straining G tolerance (SGT) under the gradual onset rate (onset rate: 0.1 G per second) (Figure 4).

**Figure 5:**
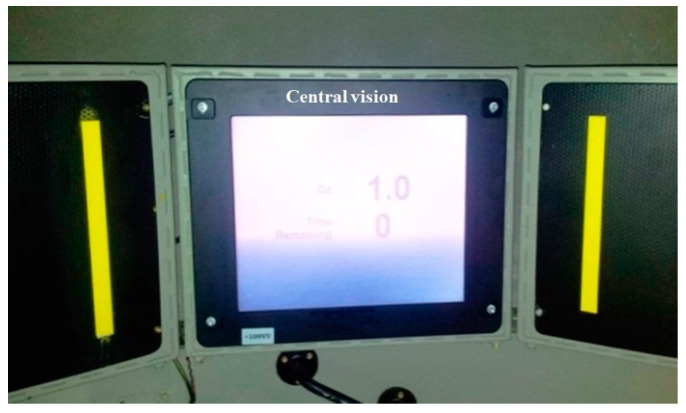
Light bars as the visual reference.

The definitions of RGT and SGT were as follows: the value of RGT was defined as the period when the participant was relaxed and experienced a 100% loss of peripheral vision or a 50% loss of central vision by using the light bars as the visual reference inside the gondola (Figure 5), and he began to perform the AGSM while G force continued to increase. The value of SGT was defined as the G level at which, after performing the AGSM, he again met the criteria for peripheral or central visual loss mentioned above and released the stick to terminate the test, or when the human centrifuge automatically smoothly braked, as controlled by the computer at the 9 G upper limit [20].

### 2.5. Data Processing of Cardiac Function

When participants finished the ground stage, we removed the BioHarness 3.0 module and exported the physiological data. We used those data recorded every second, and then stored and named them as Summary Excel files. Because there was a large amount of data, we used the method described in the report by Hsiao et al. [17] to identify when participants were resting or walking during the ground stage. Based on the BioHarness 3.0 user manual, the values of the HR confidence or system confidence lower than 20% presented that those data were unreliable and were automatically deleted [21]. In total, 94 male military aircrew were recruited to complete this study; two were excluded due to the poor data quality. Ultimately, 92 participants were entered into the analysis. Our team members further managed the physiological data collected by the BioHarness 3.0 equipment to calculate the value of the CFI variable. CFI is a well-developed method to monitor cardiac function and workload during physical activity. The advantages of CFI monitoring are that it is noninvasive and wireless and can be performed in real time by a mobile health device. The mathematical formula is CFI = weight × activity/HR [18,19]. The parameters monitored by BioHarness 3.0 were defined as follows:

1. Activity: Level and amount of activity represents the movement of the participants recorded every second by the accelerometer sensors inside the BioHarness 3.0.
2. HR: The number of beats per minute (bpm) while resting or walking was also measured by the sensor and provided as an average at the time span of one second on the BioHarness 3.0 Summary Excel file report.
3. CFI: There are two different types of CFI, resting CFI (RCFI) and walking CFI (WCFI), illustrated in this study. The average value of the RCFI was calculated by the two-minute data before the end of sit-resting status. For the WCFI, the mean value was identified by the two-minute data before the end of walking status on the ground.
4. Cardiac force ratio (CFR): The average value of the CFR was calculated using the two-minute WCFI divided by the two-minute RCFI.

### 2.6. Statistical Analysis

In the univariate analysis, we used the mean, standard deviation, max, and min to present the distribution of the continuous variables. Categorical information was displayed as numbers and percentages. The Pearson correlation coefficient was used to assess the correlation between the CFI and G tolerance.

Multiple linear regression or logistic regression with a forward selection model was applied to clarify the purpose of this study. All data were analyzed by SPSS 27.0 software (IBM, Armonk, NY, USA), and the significance level of all tests was set at the two-tailed *p* < 0.05.

## 3. Results

### 3.1. Characteristics of Study Participants

Table 1 shows the descriptive demographic data of 92 voluntary male participants who completed this study. The mean age was 26.17 ± 4.52 years, the mean height was 174.63 ± 5.69 cm, the mean weight was 74.39 ± 11.89 kg [SD], and the mean BMI was 24.35 ± 3.38 kg/m^2^. The proportions of smokers and drinkers were 21.7% and 15.2%, respectively. Nearly half of the study participants (48.9%) had regular exercise habits.

### 3.2. Physiological Responses on the Ground and during the G Tolerance Test

**Figure 6:**
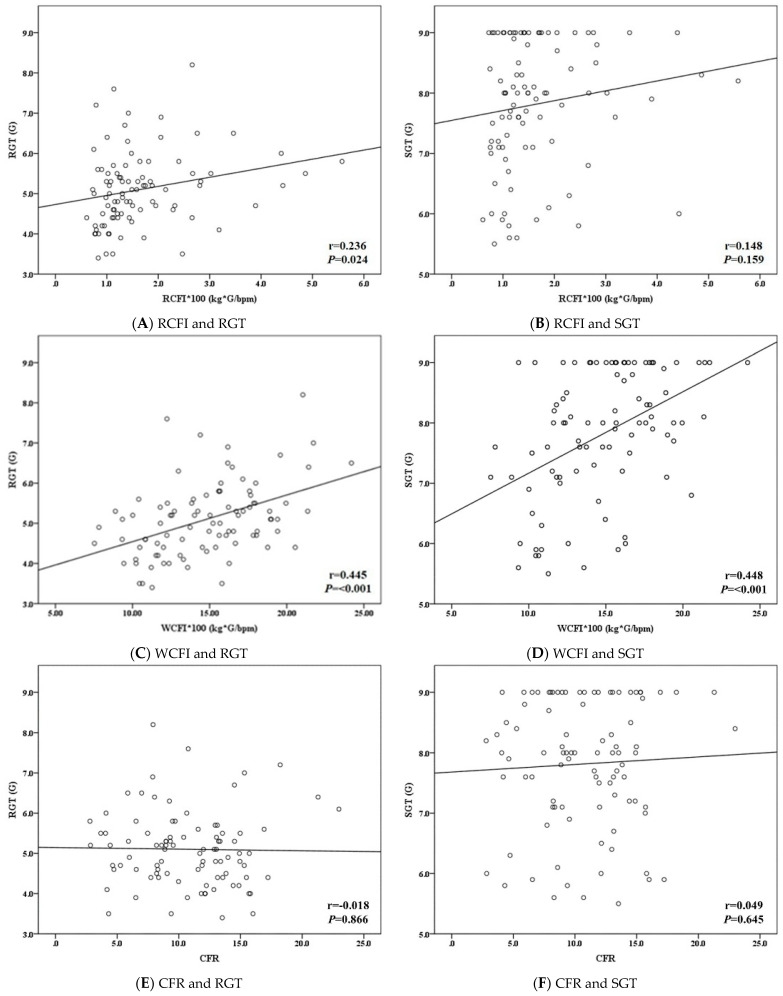
Linear association between the cardiac function and G tolerance. RCFI: resting cardiac force index; WCFI: walking cardiac force index; CFR: cardiac force ratio; RGT: relaxed G tolerance; SGT: straining G tolerance; r: Pearson correlation coefficient.

As shown in Table 2, the average values of resting SBP, DBP, and HR were 141.80 ± 13.75 mmHg, 80.78 ± 7.03 mmHg, and 84.07 ± 11.15 bmp before the G tolerance test, respectively. The mean value of the WCFI was much higher than that of the RCFI (WCFI vs. RCFI: 0.15 ± 0.04 kg × G/bpm vs. 0.02 ± 0.04 kg × G/bpm). The average CFR computed from the WCFI divided by the RCFI was 10.77 ± 4.11. The average RGT and SGT of the participants were 5.1 ± 0.9 G and 7.8 ± 1.1 G, respectively, during the G tolerance test. The percentages of participants with an RGT greater than 5 G or an SGT greater than 8 G were equally noted as 54.3%. The distribution of the RCFI was positively correlated with RGT performance (r = 0.236, *p* = 0.024). In addition, the WCFI was also significantly correlated with the RGT (r = 0.445, *p* < 0.001) and SGT (r = 0.448, *p* < 0.001), as shown in Figure 6.

### 3.3. Relationship between CFI and G Tolerance Examined by Multivariate Regression

As illustrated in Table 3, multivariate linear regression with forward selection was used to test the relationship between CFI and G tolerance. In the RGT model, each 100-unit increase in the WCFI increased the RGT by 0.14 G [SE 0.02, 95% CI 0.09 to 0.19]. Each 100 units of WCFI elevation were also positively related to the SGT [β 0.17, SE 0.03, 95% CI 0.11 to 0.22]. In Table 4, we further used the forward logistic regression method to investigate the association between the CFI and G tolerance. The results identified that the probability of an RGT greater than 5 G and an SGT greater than 8 G would increase 26% [OR = 1.26 95% CI 1.10 to 1.46] and 50% [OR = 1.50 95% CI 1.24 to 1.82] for each 100 unit increase in the WCFI.

## 4. Discussion

According to previous reports, traditional cardiovascular parameters have been widely used to establish a relationship with G tolerance in the centrifuge. In the United States Air Force (USAF), the wearable health technology of BioHarness has proven the consistency of the acceleration level during high-G flight with the aircraft system [22]. Our work was the first study to successfully introduce the CFI variable for the prediction of G tolerance among male military aircrew. The main results illustrated that walking cardiac function on the ground is positively related to G tolerance in the simulated high-G environment.

RGT, defined as a relaxed condition to tolerate G force, often ranges from 4.5 G to 6 G [23]. SGT presents the combined effect of RGT and AGSM effectiveness. Due to the loss of several high-performance aircraft caused by GLOC, the USAF School of Aerospace Medicine began high-G training in 1985. The average values of RGT and SGT of 1426 pilots were 5.2 G and 8.3 G on the gradual onset run [20]. In this study, the average values of RGT and SGT were 5.1 G and 7.8 G, respectively, among the participants. Previous results have noted that age, flight experience/hour [24,25], and repeated exposure to a high-G environment [2,9,26] were positive factors correlated with G tolerance. The mild discrepancy in SGT might well result from the different age subgroups of male military aircrew members. In the USAF study, trainees were experienced fighter pilots well trained to cope with high-G stress during flight. In contrast, our participants were recruited from among young male military aircrew who were naïve to high-G exposure.

In the correlation test, our findings showed that RCFI had only a weak relationship to RGT but no significant correlation to SGT. In contrast, there were moderate relationships between WCFI and G tolerance, including both RGT and SGT. It seemed that WCFI was a more suitable parameter for the prediction model of G tolerance. The G tolerance test in the human centrifuge is a vigorous, high-intensity, and low-interval exercise such as sprinting; moreover, trainees must perform an efficient AGSM to enhance cardiovascular function against G stress. Cardiovascular responses regulated by the autonomic nervous system play a crucial role in G tolerance. From the centrifuge training database, our team found that failed trainees had a lower proportion of increases in HR than passing trainees [10,11]. Therefore, dynamic CFI should be a much better cardiac indicator used to establish a predictive model for G tolerance. In the final multivariate model, our results also proved that WCFI had a significantly positive relation to the G tolerance level.

Military aircrew must be qualified by the different standards of high-G training courses based on the airframes. The training target of flying jet fighters is to sustain 7.5 G for 15 s. High-performance fighter pilots are required to tolerate the challenge of 9 G for 15 s, at a very high-onset rate. Previous reports revealed that military aircrew had a higher chance of failing during the 9 G–15-s training if their RGT or SGT was less than 5 G or 8 G, respectively [11,20]. In the next step, we classified study participants into different G tolerance groups that depended on RGT (≥5 G vs. <5 G) or SGT (≥8 G vs. <8 G) and distinguished the WCFI differences between those groups. After adjustment for confounders, we also identified that participants with higher WCFI had higher G tolerance during training, especially for SGT. In addition, AGSM effectiveness is one of the determinants of SGT; moreover, it compensates for the weaker cardiovascular responses against GLOC [11]. Therefore, compared to RGT, we found that the WCFI had a stronger correlation with the SGT value. In other words, the potential connection between the CFI and AGSM effectiveness is worth exploring in future work.

Hydrostatic pressure is proportional to the level of G force. A reduction in blood pressure at the brain level is induced by hydrostatic pressure. On Earth (1 G environment), there is an average reduction of 22 to 23 mmHg from the heart to the brain. Taller study participants would theoretically have an increased gradient of hydrostatic pressure during exposure to the high-G condition. Among US fighter pilots, Webb et al. [20] declared that height could have a negative relation to RGT and SGT. In the Republic of Korea Air Force, Park et al. [27] indicated that the subjects’ G tolerance duration was inversely correlated with their height. In agreement with former reports, our findings also clarified that the height of the study participants was obviously negatively connected to RGT and SGT in the final regression model. The mechanism of G tolerance determination is well known and consists of multiple layers of biological covariates. In future studies, it would be interesting to collect this information (e.g., body composition, biochemistry data, etc.) to examine the main effects and their interaction terms on G tolerance.

To manipulate modern fighters, military aircrew must tolerate G stress and perform effective AGSM by implementing a physical conditioning program. The general principles focus on the fundamentals of whole-body strength, power, endurance and mobility. Many studies have revealed that strength and weight training could increase trainees’ tolerance time in simulated acrobatic combat maneuver profiles with rapid onset rates [28,29,30,31]. Similar to those studies, our results in this study noted that participants with regular exercise habits also have the ability to withstand a high G load. However, the peak value of G tolerance enhanced by physical conditioning is still debated. In addition, the recovery ability from repeated G exposure is also one determinant of G tolerance [10,11,32]. The demand of the G tolerance test is highly intensified physical exertion and short-interval rest with fast recovery. Therefore, the contents of physical conditioning consist of more anaerobic activity and a moderate amount of aerobic sports to enhance G tolerance.

Although there were no details of exercise habits among the study participants, they had knowledge of physical fitness training for military aircrew from physical education in the early stage of their military careers. This might be a potential explanation for the finding that exercise habits were correlated only with RGT levels. In addition, physical conditioning could also improve cardiovascular function. Further work should be organized to assess the effects of physical conditioning on the CFI by stratification analysis.

There are still several limitations and weaknesses in this study. First, female military aircrew were excluded from the analysis due to the small sample size. Gender differences in the CFI were not discussed in this study. Second, to better understand their cardiac function in the early stages of their flight careers, it would be more cost-effective to conduct the G-tolerance surveillance program during potential aircrew selection. We recruited only young male military aircrew, and the generalization of these results to the aircrew population could be restricted. The extended study will be organized to recruit more participants and to examine the CFI accuracy to predict the G tolerance. Next, the WCFI of these participants was calculated from the data recorded during subjectively normal-speed walking. Variations in walking speed could influence the outcome. Larger samples of the current study were included to minimize the residual effect. Finally, a human centrifuge is continuously spun to generate a high-G environment during the G tolerance test. Participants sat inside the gondola of the centrifuge and were almost inactive when attending the test. The activity level measured by the BioHarness device reflected the movement of the human centrifuge rather than that of the study participants [20]. Thus, we could not obtain the CFI in the centrifuge. In future work, we will try to develop a new formula of cardiac function during the training to predict G tolerance.

## 5. Conclusions

In conclusion, this is the first study in which we utilized wearable and mobile technology before the centrifuge training and calculated cardiac force for military aircrew. The results revealed that the WCFI on the ground has a significant positive association with G tolerance in the centrifuge. In addition, height and exercise habits could influence G tolerance. The CFI could be used as a potential indicator during the initial surveillance of low-G tolerance aircrew members. Military aircrew can appropriately undergo the program of G tolerance enhancement and increase the effectiveness and safety of flight training.

## Figures and Tables

**Table 1:** Descriptive analysis of demographic data.

Variables	Mean ± SD/*n* (%)	Min	Max
Age (years)	26.17 ± 4.52	22	47
Male	92 (100%)		
Height (cm)	174.63 ± 5.69	162	188
Weight (kg)	74.39 ± 11.89	53	104
BMI (kg/m^2^)	24.35 ± 3.38	15.34	34.28
Smoking status			
No	72 (78.3%)		
Yes	20 (21.7%)		
Drinking status			
No	78 (84.8%)		
Yes	14 (15.2%)		
Exercise habits			
No	47 (51.1%)		
Yes	45 (48.9%)		

SD: standard deviation; BMI: body mass index.

**Table 2:** Descriptive analysis of physiological parameters.

Variables	Mean ± SD	Min	Max
SBP (mmHg)	141.80 ± 13.75	108	177
DBP (mmHg)	80.78 ± 7.03	65	104
HR (bpm)	84.07 ± 11.15	66	122
RCFI (kg × G/bpm)	0.02 ± 0.01	0.01	0.06
WCFI (kg × G/bpm)	0.15 ± 0.04	0.08	0.24
CFR	10.77 ± 4.11	2.80	22.98
RGT (G)	5.1 ± 0.9	3.4	8.2
<5 G	42 (45.7%)		
≥5 G	50 (54.3%)		
SGT (G)	7.8 ± 1.1	5.5	9.0
<8 G	42 (45.7%)		
≥8 G	50 (54.3%)		

SD: standard deviation; SBP: systolic blood pressure; DBP: diastolic blood pressure; HR: heart rate; RCFI: resting cardiac force index; WCFI: walking cardiac force index; CFR: cardiac force ratio; RGT: relaxed G tolerance; SGT: straining G tolerance.

**Table 3:** Variables associated with G tolerance analyzed by multivariate linear regression.

Model/Variables	*β*	SE	*p*	95% CI
RGT Model				
Height (cm)	−0.06	0.02	<0.001	−0.08 to −0.03
WCFI × 100 (kg × G/bpm)	0.14	0.02	<0.001	0.09 to 0.19
SGT Model				
Height (cm)	−0.08	0.02	<0.001	−0.11 to −0.05
WCFI × 100 (kg × G/bpm)	0.17	0.03	<0.001	0.11 to 0.22

SE: standard error; CI: confidence interval; RGT: relaxed G tolerance; WCFI: walking cardiac force index; SGT: straining G tolerance.

**Table 4:** Factors associated with G tolerance analyzed by multivariate logistic regression.

Model/Variables	Group	*β*	SE	*p*	OR	95% CI
RGT Model (≥5 G vs. <5 G)						
Exercise habits	yes vs. no	1.10	0.47	0.019	3.01	1.20 to 7.52
WCFI × 100 (kg × G/bpm)		0.23	0.07	0.001	1.26	1.10 to 1.46
SGT Model (≥8 G vs. <8 G)						
Height (cm)		−0.21	0.06	0.001	0.81	0.72 to 0.91
WCFI × 100 (kg × G/bpm)		0.41	0.10	<0.001	1.50	1.24 to 1.82

SE: standard error; OR: odds ratio; CI: confidence interval; RGT: relaxed G tolerance; WCFI: walking cardiac force index; SGT: straining G tolerance.

## Data Availability

The data in this study are collected and owned by Aviation Physiology Research Laboratory, Kaohsiung Armed Forces General Hospital, Gangshan Branch, Taiwan. Due to the regulations, the data cannot be shared publicly.
